# RbYb(PO_3_)_4_

**DOI:** 10.1107/S1600536812050969

**Published:** 2012-12-22

**Authors:** Jing Zhu, Hui Chen

**Affiliations:** a Department of Material Science and Engineering, Yunnan University, Kunming, Yunnan 650091, People’s Republic of China

## Abstract

Rubidium ytterbium(III) tetra­kis­(polyphosphate), RbYb(PO_3_)_4_, was synthesized by solid-state reaction. It adopts structure type IV of the *MRE*(PO_3_)_4_ (*M* = alkali metal and *RE* = rare earth metal) family of compounds. The structure is composed of a three-dimensional framework made up from double spiral polyphosphate chains parallel to [10-1] and irregular [YbO_8_] polyhedra. There are eight PO_4_ tetra­hedra in the repeat unit of the polyphosphate chains. The Rb^+^ cation is located in channels extending along [100] that are delimited by the three-dimensional framework. It is surrounded by 11 O atoms, defining an irregular polyhedron.

## Related literature

For background to applications of condensed rare earth phosphates, see: Malinowski (1989 ▶); Miyazawa *et al.* (1979 ▶). For the structures of other ytterbium phosphate compounds, see: Rghioui *et al.* (2002 ▶); Fang *et al.* (2008 ▶); Hong (1974 ▶); Jansen *et al.* (1991 ▶). For an isotypic structure, see: Zhu *et al.* (2009 ▶).

## Experimental

### 

#### Crystal data

RbYb(PO_3_)_4_
*M* *_r_* = 574.40
Monoclinic, 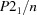

*a* = 10.2022 (15) Å
*b* = 8.7975 (13) Å
*c* = 10.9300 (16) Å
β = 106.323 (2)°
*V* = 941.5 (2) Å^3^
*Z* = 4
Mo *K*α radiation
μ = 15.80 mm^−1^
*T* = 296 K
0.10 × 0.07 × 0.04 mm

#### Data collection

Bruker APEXII CCD diffractometer
Absorption correction: multi-scan (*SADABS*; Bruker, 2008 ▶) *T*_min_ = 0.725, *T*_max_ = 0.854
9912 measured reflections
2676 independent reflections
2024 reflections with *I* > 2σ(*I*)
*R*_int_ = 0.143

#### Refinement

*R*[*F* ^2^ > 2σ(*F* ^2^)] = 0.043
*wR*(*F* ^2^) = 0.078
*S* = 1.00
2676 reflections
163 parameters
Δρ_max_ = 2.95 e Å^−3^
Δρ_min_ = −2.99 e Å^−3^

### 

Data collection: *APEX2* (Bruker, 2008 ▶); cell refinement: *SAINT* (Bruker, 2008 ▶); data reduction: *SAINT*; program(s) used to solve structure: *SHELXS97* (Sheldrick, 2008 ▶); program(s) used to refine structure: *SHELXL97* (Sheldrick, 2008 ▶); molecular graphics: *SHELXTL* (Sheldrick, 2008 ▶) and *DIAMOND* (Brandenburg, 2006 ▶); software used to prepare material for publication: *publCIF* (Westrip, 2010 ▶).

## Supplementary Material

Crystal structure: contains datablock(s) I, global. DOI: 10.1107/S1600536812050969/wm2707sup1.cif

Structure factors: contains datablock(s) I. DOI: 10.1107/S1600536812050969/wm2707Isup2.hkl

Additional supplementary materials:  crystallographic information; 3D view; checkCIF report

## Figures and Tables

**Fig. 1.**
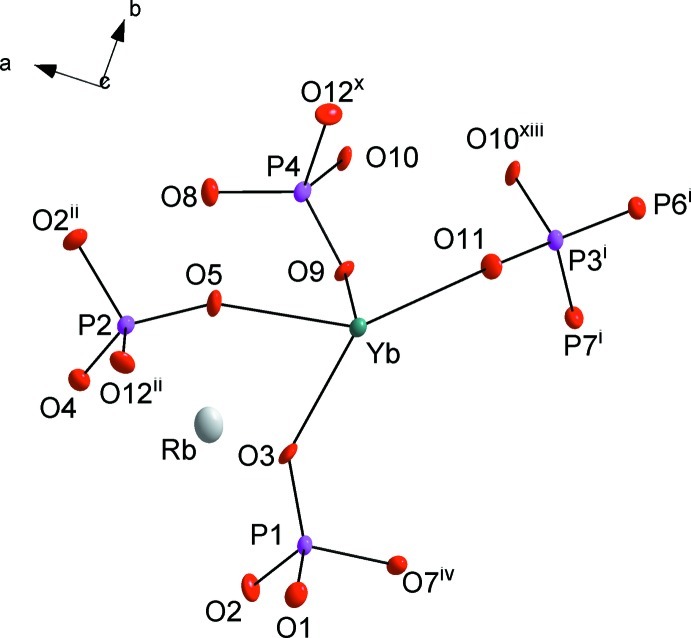
View of a part of the title structure. Ellipsoids are shown at the 50% probability level. [Symmetry code: (i) -0.5 + x, 0.5 - y, -0.5 + z; (ii) 1.5 - x, 0.5 + y, 1.5 - z; (iv) 1 - x, -y, 2 - z; (x) x, 1 + y, z; (xiii) 1 - x, 1 - y, 2 - z;]

**Fig. 2.**
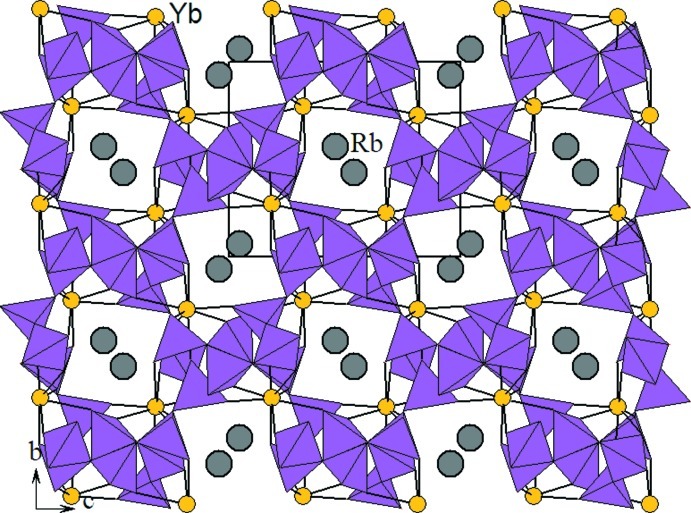
The packing of the structure of RbYb(PO3)4, viewed along the a axis. Polyhedra represents PO4 tetrahedra.

**Fig. 3.**
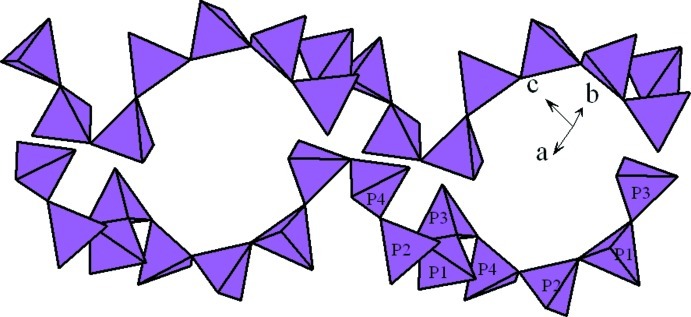
The polyphosphate double spiral chains

## supplementary crystallographic information

### Comment

Extensive studies on structures and properties of condensed rare earth phosphates have been carried out in the past owing to their potential application in the optics domain (Miyazawa *et al.*, 1979; Malinowski *et al.*, 1989). Furthermore, their chemical and thermal stability ensures the feasibility of possible applications. Numerous rare-earth phosphates with ytterbium, such as YbP_3_O_9_ (Hong, 1974), CsYbP_2_O_7_ (Jansen *et al.*, 1991), and K_2_CsYb(PO_4_)_2_ (Rghioui *et al.*, 2002) have been synthesized and structurally determined. However, the literature shows that in the polyphosphate family with general formula *MRE*(PO_3_)_4_ (*M* = monovalent cation, *RE* = rare earth cation), only a few examples with ytterbium are known [LiYb(PO_3_)_4_ (Fang *et al.*, 2008)]. The reason for this situation is probably the difficulty in obtaining crystals of high quality. Accordingly, our research group is paying attention to the preparation of new polyphosphates *M*Yb(PO_3_)_4_. We successfully grew single crystals of rubidium ytterbium polyphosphate, RbYb(PO_3_)_4_, the structure of which is reported here.

The crystal structure of RbYb(PO_3_)_4_ is shown in Figs. 1 and 2. It is isostructural with CsEu(PO_3_)_4_ (Zhu *et al.*, 2009) and belongs to type IV of the *M*RE(PO_3_)_4_ (*M* = alkali metal and RE = rare earth) family of compounds. The structure can be described as a three-dimensional framework made up from polyphosphate double spiral chains extending parallel to [101] and YbO_8_ polyhedra. The Rb^+^ cations are located in infinite tunnels along [100] delimited by the three-dimensional framework. As illustrated in Fig. 3, the P atom is four-coordinated, and four crystallographically distinct PO_4_ tetrahedra form the (PO_3_)_∞_^-^ double spiral chains by corner-sharing. The P–O bond lengths and O—P—O bond angles show normal values for catena-polyphosphates. There are eight PO_4_ tetrahedra in the repeating unit of the double spiral chain. The Yb^III^ cation is eight-coordinated in form of a distorted polyhedron with Y—O bond lengths ranging from 2.253 (5) to 2.412 (5) Å, which are consistent with those reported previously (Fang *et al.*, 2008). The shortest Yb···Yb contact is 6.3540 (8) Å. The Rb^+^ cation are located in the intersecting channels and are surrounded by eleven O atoms, with Rb—O bond lengths in the range of 2.915 (5)–3.504 (5) Å. Neighboring two RbO_11_ polyhedra are connected by corner-sharing.

### Experimental

Single crystals of RbYb(PO_3_)_4_ were grown by solid state reactions. All reagents were purchased commercially and used without further purification. The starting materials RbNO_3_, Yb_2_O_3_ and NH_4_H_2_PO_4_ were weighed in the molar ratio of Rb/Yb/P = 7/1/18 and finely ground in an agate mortar to ensure the best homogeneity and reactivity, and were then placed in a corundum crucible and preheated at 373 K for 6 h. Afterwards, the material was reground and heated to 723 K for 36 h and then cooled to 393 K at a rate of 6 K/h and finally air-quenched to room temperature. A few colourless block-shaped crystals were obtained from the reaction product.

### Refinement

EDX spectrometry using a JSM6700F scanning electron microscope confirmed the composition. The remaining maximum and minimum electron densities are located 0.87 Å and 0.81 Å, respectively, from the Rb atom.

### Crystal data

**Table:**

RbYb(PO_3_)_4_	*F*(000) = 1049
*M**_r_* = 574.40	*D*_x_ = 4.052 Mg m^−^^3^
Monoclinic, *P*2_1_/*n*	Mo *K*α radiation, λ = 0.71073 Å
Hall symbol: -P 2yn	Cell parameters from 2802 reflections
*a* = 10.2022 (15) Å	θ = 2.4–29.5°
*b* = 8.7975 (13) Å	µ = 15.80 mm^−^^1^
*c* = 10.9300 (16) Å	*T* = 296 K
β = 106.323 (2)°	Block, colorless
*V* = 941.5 (2) Å^3^	0.10 × 0.07 × 0.04 mm
*Z* = 4	

### Data collection

**Table:**

Bruker APEXII CCD diffractometer	2676 independent reflections
Radiation source: fine-focus sealed tube	2024 reflections with *I* > 2σ(*I*)
Graphite monochromator	*R*_int_ = 0.143
φ and ω scans	θ_max_ = 30.1°, θ_min_ = 2.4°
Absorption correction: multi-scan (*SADABS*; Bruker, 2008)	*h* = −14→14
*T*_min_ = 0.725, *T*_max_ = 0.854	*k* = −12→12
9912 measured reflections	*l* = −15→15

### Refinement

**Table:**

Refinement on *F*^2^	0 restraints
Least-squares matrix: full	Primary atom site location: structure-invariant direct methods
*R*[*F*^2^ > 2σ(*F*^2^)] = 0.043	Secondary atom site location: difference Fourier map
*wR*(*F*^2^) = 0.078	*w* = 1/[σ^2^(*F*_o_^2^) + (0.016*P*)^2^] where *P* = (*F*_o_^2^ + 2*F*_c_^2^)/3
*S* = 1.00	(Δ/σ)_max_ < 0.001
2676 reflections	Δρ_max_ = 2.95 e Å^−^^3^
163 parameters	Δρ_min_ = −2.99 e Å^−^^3^

### Special details

**Table:**

Geometry. All e.s.d.'s (except the e.s.d. in the dihedral angle between two l.s. planes) are estimated using the full covariance matrix. The cell e.s.d.'s are taken into account individually in the estimation of e.s.d.'s in distances, angles and torsion angles; correlations between e.s.d.'s in cell parameters are only used when they are defined by crystal symmetry. An approximate (isotropic) treatment of cell e.s.d.'s is used for estimating e.s.d.'s involving l.s. planes.
Refinement. Refinement of *F*^2^ against ALL reflections. The weighted *R*-factor *wR* and goodness of fit *S* are based on *F*^2^, conventional *R*-factors *R* are based on *F*, with *F* set to zero for negative *F*^2^. The threshold expression of *F*^2^ > σ(*F*^2^) is used only for calculating *R*-factors(gt) *etc*. and is not relevant to the choice of reflections for refinement. *R*-factors based on *F*^2^ are statistically about twice as large as those based on *F*, and *R*- factors based on ALL data will be even larger.

### Fractional atomic coordinates and isotropic or equivalent isotropic displacement parameters (Å^2^)

**Table:**

	*x*	*y*	*z*	*U*_iso_*/*U*_eq_	
Yb	0.49845 (3)	0.22753 (3)	0.68227 (3)	0.00898 (9)	
Rb	0.68841 (9)	−0.06519 (10)	0.95773 (7)	0.02424 (19)	
P1	0.45844 (18)	−0.1715 (2)	0.63271 (16)	0.0088 (3)	
P2	0.85263 (18)	0.0916 (2)	0.74217 (16)	0.0093 (3)	
P3	0.75504 (18)	0.0245 (2)	1.27947 (15)	0.0088 (3)	
P4	0.67393 (18)	0.3877 (2)	0.97599 (16)	0.0091 (3)	
O1	0.4366 (5)	−0.2434 (6)	0.5063 (4)	0.0135 (11)	
O2	0.5242 (5)	−0.2914 (6)	0.7444 (5)	0.0141 (10)	
O3	0.5348 (5)	−0.0274 (6)	0.6642 (5)	0.0144 (10)	
O4	0.8988 (5)	−0.0414 (6)	0.8251 (5)	0.0137 (10)	
O5	0.7325 (5)	0.1773 (6)	0.7555 (5)	0.0142 (10)	
O6	0.6458 (5)	−0.0858 (6)	1.2179 (4)	0.0112 (10)	
O7	0.6895 (5)	0.1572 (6)	1.3448 (5)	0.0122 (10)	
O8	0.8156 (5)	0.3283 (6)	1.0176 (5)	0.0140 (11)	
O9	0.5637 (5)	0.2853 (6)	0.9024 (4)	0.0118 (10)	
O10	0.6370 (5)	0.4504 (6)	1.1006 (4)	0.0100 (10)	
O11	0.3317 (5)	0.4059 (6)	0.6976 (4)	0.0125 (10)	
O12	0.6715 (5)	−0.4534 (6)	0.9034 (4)	0.0134 (10)	

### Atomic displacement parameters (Å^2^)

**Table:**

	*U*^11^	*U*^22^	*U*^33^	*U*^12^	*U*^13^	*U*^23^
Yb	0.00774 (14)	0.01101 (15)	0.00853 (13)	−0.00035 (12)	0.00286 (9)	−0.00056 (12)
Rb	0.0245 (4)	0.0340 (5)	0.0145 (4)	0.0045 (3)	0.0060 (3)	−0.0013 (3)
P1	0.0063 (8)	0.0108 (8)	0.0098 (8)	0.0007 (6)	0.0029 (6)	0.0001 (7)
P2	0.0078 (8)	0.0108 (9)	0.0089 (8)	−0.0006 (6)	0.0018 (6)	−0.0012 (6)
P3	0.0074 (8)	0.0117 (9)	0.0076 (8)	−0.0011 (6)	0.0024 (7)	0.0015 (6)
P4	0.0076 (8)	0.0127 (9)	0.0066 (7)	−0.0003 (6)	0.0015 (6)	0.0006 (6)
O1	0.013 (2)	0.020 (3)	0.009 (2)	−0.001 (2)	0.0051 (18)	−0.0006 (19)
O2	0.012 (2)	0.018 (3)	0.011 (2)	0.006 (2)	0.0026 (19)	0.006 (2)
O3	0.006 (2)	0.016 (3)	0.022 (3)	−0.005 (2)	0.005 (2)	−0.002 (2)
O4	0.014 (3)	0.013 (3)	0.014 (2)	0.001 (2)	0.005 (2)	−0.002 (2)
O5	0.006 (2)	0.021 (3)	0.015 (2)	0.002 (2)	0.0018 (19)	−0.005 (2)
O6	0.010 (2)	0.013 (3)	0.011 (2)	−0.0024 (19)	0.0025 (19)	−0.0004 (19)
O7	0.011 (2)	0.012 (3)	0.015 (2)	−0.002 (2)	0.006 (2)	−0.0038 (19)
O8	0.010 (2)	0.020 (3)	0.013 (2)	0.004 (2)	0.0054 (19)	0.002 (2)
O9	0.007 (2)	0.015 (2)	0.011 (2)	−0.004 (2)	0.0002 (18)	−0.003 (2)
O10	0.005 (2)	0.019 (3)	0.007 (2)	−0.0004 (19)	0.0035 (18)	−0.0024 (19)
O11	0.014 (3)	0.017 (3)	0.010 (2)	0.001 (2)	0.010 (2)	0.001 (2)
O12	0.019 (3)	0.013 (3)	0.007 (2)	−0.002 (2)	0.002 (2)	0.0045 (19)

### Geometric parameters (Å, º)

**Table:**

Yb—O8^i^	2.253 (5)	P2—O4	1.474 (5)
Yb—O3	2.291 (5)	P2—O5	1.480 (5)
Yb—O4^ii^	2.300 (5)	P2—O12^ii^	1.590 (5)
Yb—O1^iii^	2.339 (5)	P2—O2^ii^	1.599 (5)
Yb—O5	2.337 (5)	P2—Rb^ii^	3.681 (2)
Yb—O11	2.355 (5)	P3—O11^vi^	1.477 (5)
Yb—O9	2.364 (5)	P3—O6	1.488 (5)
Yb—O6^iv^	2.412 (5)	P3—O10^vii^	1.595 (5)
Yb—P4	3.5025 (18)	P3—O7	1.609 (5)
Yb—P3^iv^	3.5196 (18)	P3—Yb^iv^	3.5196 (18)
Yb—Rb	4.0215 (9)	P4—O8	1.483 (5)
Yb—Rb^ii^	4.3152 (10)	P4—O9	1.489 (5)
Rb—O4	2.915 (5)	P4—O12^x^	1.604 (5)
Rb—O1^v^	2.962 (5)	P4—O10	1.609 (5)
Rb—O11^vi^	2.970 (5)	O1—Yb^iii^	2.339 (5)
Rb—O6	3.000 (5)	O1—Rb^ix^	2.962 (5)
Rb—O2	3.164 (5)	O2—P2^viii^	1.599 (5)
Rb—O3	3.168 (5)	O4—Yb^viii^	2.300 (5)
Rb—O5	3.193 (5)	O5—Rb^ii^	3.504 (5)
Rb—O7^vii^	3.265 (5)	O6—Yb^iv^	2.412 (5)
Rb—O9	3.326 (5)	O7—P1^iv^	1.601 (5)
Rb—O12	3.463 (5)	O7—Rb^xi^	3.265 (5)
Rb—P3	3.4796 (19)	O8—Yb^vi^	2.253 (5)
Rb—O5^viii^	3.504 (5)	O10—P3^xi^	1.595 (5)
P1—O3	1.477 (5)	O11—P3^i^	1.477 (5)
P1—O1	1.479 (5)	O11—Rb^i^	2.970 (5)
P1—O7^iv^	1.601 (5)	O12—P2^viii^	1.590 (5)
P1—O2	1.610 (5)	O12—P4^xii^	1.604 (5)
P1—Rb^ix^	3.695 (2)		
			
O8^i^—Yb—O3	80.51 (18)	O6—Rb—P3	25.18 (10)
O8^i^—Yb—O4^ii^	116.63 (18)	O2—Rb—P3	143.14 (10)
O3—Yb—O4^ii^	140.91 (18)	O3—Rb—P3	154.20 (11)
O8^i^—Yb—O1^iii^	71.65 (18)	O5—Rb—P3	121.50 (10)
O3—Yb—O1^iii^	83.67 (18)	O7^vii^—Rb—P3	64.64 (9)
O4^ii^—Yb—O1^iii^	70.81 (17)	O9—Rb—P3	85.96 (9)
O8^i^—Yb—O5	140.30 (18)	O12—Rb—P3	112.54 (9)
O3—Yb—O5	70.73 (18)	O4—Rb—O5^viii^	51.42 (13)
O4^ii^—Yb—O5	75.36 (18)	O1^v^—Rb—O5^viii^	53.59 (12)
O1^iii^—Yb—O5	78.36 (18)	O11^vi^—Rb—O5^viii^	138.09 (13)
O8^i^—Yb—O11	75.48 (18)	O6—Rb—O5^viii^	135.50 (13)
O3—Yb—O11	142.76 (18)	O2—Rb—O5^viii^	43.63 (12)
O4^ii^—Yb—O11	76.06 (17)	O3—Rb—O5^viii^	62.28 (13)
O1^iii^—Yb—O11	114.32 (16)	O5—Rb—O5^viii^	82.41 (2)
O5—Yb—O11	142.25 (18)	O7^vii^—Rb—O5^viii^	80.78 (12)
O8^i^—Yb—O9	142.46 (17)	O9—Rb—O5^viii^	128.79 (12)
O3—Yb—O9	107.00 (18)	O12—Rb—O5^viii^	42.07 (12)
O4^ii^—Yb—O9	81.08 (17)	P3—Rb—O5^viii^	143.41 (9)
O1^iii^—Yb—O9	144.61 (17)	O3—P1—O1	121.0 (3)
O5—Yb—O9	73.97 (17)	O3—P1—O7^iv^	110.8 (3)
O11—Yb—O9	77.69 (17)	O1—P1—O7^iv^	106.0 (3)
O8^i^—Yb—O6^iv^	77.00 (17)	O3—P1—O2	107.8 (3)
O3—Yb—O6^iv^	70.55 (17)	O1—P1—O2	110.4 (3)
O4^ii^—Yb—O6^iv^	144.63 (17)	O7^iv^—P1—O2	98.3 (3)
O1^iii^—Yb—O6^iv^	142.15 (17)	O3—P1—Rb^ix^	157.9 (2)
O5—Yb—O6^iv^	116.22 (17)	O1—P1—Rb^ix^	49.7 (2)
O11—Yb—O6^iv^	76.54 (17)	O7^iv^—P1—Rb^ix^	62.01 (18)
O9—Yb—O6^iv^	71.55 (16)	O2—P1—Rb^ix^	94.2 (2)
O8^i^—Yb—P4	156.26 (13)	O3—P1—Rb	54.7 (2)
O3—Yb—P4	114.82 (13)	O1—P1—Rb	150.9 (2)
O4^ii^—Yb—P4	63.63 (12)	O7^iv^—P1—Rb	101.48 (18)
O1^iii^—Yb—P4	125.81 (12)	O2—P1—Rb	55.5 (2)
O5—Yb—P4	63.41 (13)	Rb^ix^—P1—Rb	144.73 (6)
O11—Yb—P4	81.96 (12)	O4—P2—O5	118.2 (3)
O9—Yb—P4	19.19 (12)	O4—P2—O12^ii^	110.5 (3)
O6^iv^—Yb—P4	90.74 (11)	O5—P2—O12^ii^	109.0 (3)
O8^i^—Yb—P3^iv^	59.09 (13)	O4—P2—O2^ii^	110.3 (3)
O3—Yb—P3^iv^	62.33 (13)	O5—P2—O2^ii^	108.4 (3)
O4^ii^—Yb—P3^iv^	156.75 (13)	O12^ii^—P2—O2^ii^	98.6 (3)
O1^iii^—Yb—P3^iv^	122.81 (12)	O4—P2—Rb	53.8 (2)
O5—Yb—P3^iv^	123.65 (14)	O5—P2—Rb	64.7 (2)
O11—Yb—P3^iv^	80.89 (13)	O12^ii^—P2—Rb	126.9 (2)
O9—Yb—P3^iv^	91.18 (12)	O2^ii^—P2—Rb	134.3 (2)
O6^iv^—Yb—P3^iv^	19.64 (11)	O4—P2—Rb^ii^	168.4 (2)
P4—Yb—P3^iv^	110.36 (4)	O5—P2—Rb^ii^	71.5 (2)
O8^i^—Yb—Rb	125.11 (14)	O12^ii^—P2—Rb^ii^	69.6 (2)
O3—Yb—Rb	51.84 (13)	O2^ii^—P2—Rb^ii^	58.8 (2)
O4^ii^—Yb—Rb	117.65 (12)	Rb—P2—Rb^ii^	136.06 (6)
O1^iii^—Yb—Rb	120.11 (13)	O11^vi^—P3—O6	116.9 (3)
O5—Yb—Rb	52.52 (13)	O11^vi^—P3—O10^vii^	107.9 (3)
O11—Yb—Rb	125.40 (11)	O6—P3—O10^vii^	111.2 (3)
O9—Yb—Rb	55.76 (13)	O11^vi^—P3—O7	109.0 (3)
O6^iv^—Yb—Rb	63.71 (11)	O6—P3—O7	108.8 (3)
P4—Yb—Rb	63.69 (3)	O10^vii^—P3—O7	102.0 (3)
P3^iv^—Yb—Rb	73.93 (3)	O11^vi^—P3—Rb	57.8 (2)
O8^i^—Yb—Rb^ii^	109.77 (13)	O6—P3—Rb	59.07 (19)
O3—Yb—Rb^ii^	103.25 (13)	O10^vii^—P3—Rb	129.2 (2)
O4^ii^—Yb—Rb^ii^	39.06 (13)	O7—P3—Rb	128.73 (19)
O1^iii^—Yb—Rb^ii^	40.64 (12)	O11^vi^—P3—Yb^iv^	149.9 (2)
O5—Yb—Rb^ii^	54.17 (13)	O6—P3—Yb^iv^	33.00 (19)
O11—Yb—Rb^ii^	111.43 (12)	O10^vii^—P3—Yb^iv^	90.31 (19)
O9—Yb—Rb^ii^	104.20 (12)	O7—P3—Yb^iv^	89.63 (19)
O6^iv^—Yb—Rb^ii^	170.36 (11)	Rb—P3—Yb^iv^	92.05 (4)
P4—Yb—Rb^ii^	85.20 (3)	O8—P4—O9	118.4 (3)
P3^iv^—Yb—Rb^ii^	161.86 (3)	O8—P4—O12^x^	109.7 (3)
Rb—Yb—Rb^ii^	106.683 (15)	O9—P4—O12^x^	110.8 (3)
O4—Rb—O1^v^	54.42 (14)	O8—P4—O10	107.5 (3)
O4—Rb—O11^vi^	99.00 (14)	O9—P4—O10	110.1 (3)
O1^v^—Rb—O11^vi^	85.77 (14)	O12^x^—P4—O10	98.4 (3)
O4—Rb—O6	142.98 (13)	O8—P4—Yb	110.0 (2)
O1^v^—Rb—O6	98.05 (13)	O9—P4—Yb	31.48 (19)
O11^vi^—Rb—O6	50.08 (14)	O12^x^—P4—Yb	87.94 (19)
O4—Rb—O2	89.44 (14)	O10—P4—Yb	137.13 (18)
O1^v^—Rb—O2	91.34 (13)	O8—P4—Rb	67.5 (2)
O11^vi^—Rb—O2	167.11 (13)	O9—P4—Rb	53.3 (2)
O6—Rb—O2	118.21 (14)	O12^x^—P4—Rb	147.1 (2)
O4—Rb—O3	73.31 (13)	O10—P4—Rb	113.9 (2)
O1^v^—Rb—O3	113.31 (13)	Yb—P4—Rb	64.49 (3)
O11^vi^—Rb—O3	145.61 (14)	P1—O1—Yb^iii^	142.4 (3)
O6—Rb—O3	143.52 (13)	P1—O1—Rb^ix^	107.9 (2)
O2—Rb—O3	46.43 (13)	Yb^iii^—O1—Rb^ix^	108.41 (17)
O4—Rb—O5	48.79 (13)	P2^viii^—O2—P1	129.8 (3)
O1^v^—Rb—O5	102.94 (13)	P2^viii^—O2—Rb	95.6 (2)
O11^vi^—Rb—O5	99.53 (14)	P1—O2—Rb	99.7 (2)
O6—Rb—O5	141.49 (14)	P1—O3—Yb	140.6 (3)
O2—Rb—O5	93.36 (13)	P1—O3—Rb	102.9 (3)
O3—Rb—O5	49.81 (13)	Yb—O3—Rb	93.51 (16)
O4—Rb—O7^vii^	100.66 (14)	P2—O4—Yb^viii^	138.6 (3)
O1^v^—Rb—O7^vii^	46.25 (13)	P2—O4—Rb	102.2 (2)
O11^vi^—Rb—O7^vii^	76.57 (13)	Yb^viii^—O4—Rb	111.14 (19)
O6—Rb—O7^vii^	57.20 (13)	P2—O5—Yb	149.0 (3)
O2—Rb—O7^vii^	92.37 (13)	P2—O5—Rb	90.6 (2)
O3—Rb—O7^vii^	137.41 (14)	Yb—O5—Rb	91.97 (16)
O5—Rb—O7^vii^	148.80 (13)	P2—O5—Rb^ii^	84.9 (2)
O4—Rb—O9	98.07 (14)	Yb—O5—Rb^ii^	93.09 (16)
O1^v^—Rb—O9	145.46 (13)	Rb—O5—Rb^ii^	174.93 (17)
O11^vi^—Rb—O9	77.95 (13)	P3—O6—Yb^iv^	127.4 (3)
O6—Rb—O9	94.50 (13)	P3—O6—Rb	95.7 (2)
O2—Rb—O9	110.67 (12)	Yb^iv^—O6—Rb	136.85 (19)
O3—Rb—O9	70.33 (13)	P1^iv^—O7—P3	130.7 (3)
O5—Rb—O9	51.39 (12)	P1^iv^—O7—Rb^xi^	92.3 (2)
O7^vii^—Rb—O9	150.34 (12)	P3—O7—Rb^xi^	135.0 (2)
O4—Rb—O12	89.65 (13)	P4—O8—Yb^vi^	147.0 (3)
O1^v^—Rb—O12	57.93 (13)	P4—O9—Yb	129.3 (3)
O11^vi^—Rb—O12	127.26 (12)	P4—O9—Rb	105.7 (2)
O6—Rb—O12	95.14 (13)	Yb—O9—Rb	88.25 (15)
O2—Rb—O12	42.48 (12)	P3^xi^—O10—P4	124.3 (3)
O3—Rb—O12	86.74 (13)	P3^i^—O11—Yb	146.7 (3)
O5—Rb—O12	123.37 (12)	P3^i^—O11—Rb^i^	97.3 (2)
O7^vii^—Rb—O12	50.74 (12)	Yb—O11—Rb^i^	115.97 (17)
O9—Rb—O12	152.28 (12)	P2^viii^—O12—P4^xii^	133.6 (3)
O4—Rb—P3	121.61 (10)	P2^viii^—O12—Rb	84.9 (2)
O1^v^—Rb—P3	91.92 (10)	P4^xii^—O12—Rb	141.4 (2)
O11^vi^—Rb—P3	24.90 (10)		

Symmetry codes: (i) *x*−1/2, −*y*+1/2, *z*−1/2; (ii) −*x*+3/2, *y*+1/2, −*z*+3/2; (iii) −*x*+1, −*y*, −*z*+1; (iv) −*x*+1, −*y*, −*z*+2; (v) *x*+1/2, −*y*−1/2, *z*+1/2; (vi) *x*+1/2, −*y*+1/2, *z*+1/2; (vii) −*x*+3/2, *y*−1/2, −*z*+5/2; (viii) −*x*+3/2, *y*−1/2, −*z*+3/2; (ix) *x*−1/2, −*y*−1/2, *z*−1/2; (x) *x*, *y*+1, *z*; (xi) −*x*+3/2, *y*+1/2, −*z*+5/2; (xii) *x*, *y*−1, *z*.
